# A Radar Waveform Design Method Based on Multicarrier Phase Coding for Suppressing Autocorrelation Sidelobes

**DOI:** 10.3390/s25185801

**Published:** 2025-09-17

**Authors:** Ji Li, Liu Ye, Wei Wang

**Affiliations:** School of Physics and Electronic Science, Changsha University of Science and Technology, Changsha 410114, China; yeliu122001@163.com (L.Y.); wangwei@csust.edu.cn (W.W.)

**Keywords:** chaos coding, genetic algorithm, time sidelobes suppression, MCPC signal, radar waveform design

## Abstract

Multicarrier phase-coded radar waveforms show significant potential in broadband radar applications by integrating phase coding with orthogonal frequency division multiplexing (OFDM) technology. However, their inherent high autocorrelation sidelobe levels limit system performance. To address this challenge, this paper proposes a two-stage joint optimization waveform design method. In the first stage, we construct an AC-MCPC signal by introducing chaotic coding in the time domain and applying a hamming window in the frequency domain, achieving effective sidelobe suppression. In the second stage, to achieve even lower sidelobe levels, we further propose the AC-MCPC-g signal. While retaining chaotic coding in the time domain, we employ a genetic algorithm in the frequency domain to optimize the window function parameters, thereby further reducing the sidelobe levels of the AC-MCPC signal. The results indicate that the AC-MCPC signal has significantly reduced sidelobes compared to the MCPC signal, while the AC-MCPC-g signal has achieved further suppression based on the AC-MCPC.

## 1. Introduction

Radar, as a fundamental electronic device for spatial detection, derives its utility from the analyzing of electromagnetic wave echoes reflected from targets. This process enables the precise determination of target parameters, including range, velocity, and angle, forming the foundation of numerous modern sensing and surveillance applications. As the operational environments for radar systems become increasingly complex, the performance requirements for radar waveforms have correspondingly escalated. In response to these demands, multicarrier phase coding (MCPC) waveforms have been proposed as a significant advancement in broadband signal design [1]. This approach synergistically combines phase coding with orthogonal frequency division multiplexing (OFDM) technology, implementing phase modulation across a bank of subcarriers. This integration confers substantial advantages, notably a marked improvement in range resolution and enhanced resilience against electronic jamming, thereby elevating the overall capability of contemporary radar systems. Despite these merits, a critical limitation of MCPC waveforms is the high peak sidelobe level (PSL) and elevated sidelobe level (SLL) inherent in their autocorrelation function following pulse compression. These undesirable sidelobe characteristics pose a significant threat to radar performance, as they can compress the system’s dynamic range, mask the returns from closely spaced targets, and degrade the signal-to-clutter ratio for detecting weak targets. Therefore, developing effective techniques for sidelobe suppression is not merely an optimization task but a critical challenge that must be addressed to fully realize the potential of MCPC waveform design.

Addressing this challenge requires a multi-faceted approach, considering the entire radar signal chain. At the transmitter, suppression can be achieved through meticulous waveform design, while at the receiver, the optimization of mismatched filters offers a potent means to reduce sidelobes [2]. Furthermore, the problem is not confined to the waveform alone; the final observed SLL is also critically dependent on the configuration of the transmitter array and the direction of arrival of the target [3]. This underscores the importance of antenna design, where optimization algorithms are employed to modify the array structure and achieve low SLL. The efficacy of such antennas is well-established, as their ability to minimize sidelobe gain directly reduces interference, thereby enhancing the signal detection capability of the main lobe and improving the radar’s overall ability to receive target signals [4].

A variety of methods have been proposed in existing research to address the issue of sidelobe suppression. In [5], the $L_{p}$-norm metric is employed to jointly design the transmit waveform and mismatched filter, but this approach suffers from high computational complexity. In [6], the concept of a virtual antenna array is utilized to reduce the sidelobes of a uniform planar array, but it is subject to limitations in element spacing. In [7], sidelobe suppression in the structure of a tapered planar array is investigated, yet the array design process is relatively complex. In terms of waveform design, the impact of circular shifts in binary sequences on the autocorrelation sidelobes of M-sequences is analyzed in [8], a gradient-based algorithm for designing low autocorrelation sequences is proposed in [9], the ADMM method is applied for power-constrained waveform design in [10], and the importance of designing well-correlated waveform sequences to reduce PSL is emphasized in [11]. Additionally, an MVDR beamformer based on a larger virtual aperture array is designed in [12], low sidelobes are achieved by optimizing the array structure and phase distribution in [13], adaptive pulse compression techniques for MIMO radar are studied in [14], and a developed adaptive beamforming algorithm realizes significant SLL improvement in [15]. Although these methods have their own emphases, such as exploring joint optimization of initial phase and bandwidth or employing complementary amplitude coding in [16] and [17], they either have high equipment complexity, limited applicability, or difficulty in directly optimizing the sidelobe performance of pulse compression for MCPC waveforms.

Nevertheless, a critical distinction exists between existing sidelobe suppression strategies and our proposed methodology. Conventional approaches, such as minimum variance distortionless response (MVDR) beamforming [18], are fundamentally rooted in array signal processing. The emphasis of MVDR is on spatial-domain interference mitigation; it operates by independently suppressing sidelobe blocking interference at the receiver and mainlobe deceptive interference at the transmitter to bolster the radar’s anti-jamming capabilities. In the pursuit of sidelobe suppression, several advanced optimization strategies have been developed, each with distinct theoretical underpinnings and practical implications. The alternating direction method of multipliers (ADMM) stands out as a powerful distributed algorithm for convex optimization, adept at solving the constrained optimization problems inherent in MIMO waveform design. Its primary advantage lies in its computational efficiency when tackling large-scale problems, though this efficacy is contingent upon the problem’s convexity. Chaotic coding exploits the inherent randomness and aperiodicity of chaotic signals, generating sequences with favorable sidelobe characteristics via iterative mapping. Similarly, genetic algorithms, a class of heuristic optimization methods, simulate the principles of natural selection to navigate the vast parameter space in search of optimal waveform weights, thereby minimizing sidelobe peaks. Our approach, by contrast, pivots from the spatial domain to the signal processing domain. We introduce a synergistic framework that jointly optimizes time-domain coding and frequency-domain windowing. This paradigm allows for a substantial reduction in the autocorrelation sidelobe level while maintaining a low computational burden. Consequently, our method presents a compelling alternative for applications where the simplicity and computational tractability of the solution are paramount.

A fundamental trade-off in radar waveform design is the improvement of range resolution, typically achieved through pulse compression. However, this enhancement often results in elevated range sidelobes [19], which pose a significant challenge to radar performance. When these sidelobes are sufficiently high, they can obscure faint echoes from weak targets, leading to missed detections and thereby compromising the system’s overall performance and reliability. To address this critical limitation, we propose leveraging orthogonal frequency division multiplexing (OFDM) technology. OFDM is an advanced multicarrier modulation scheme that partitions a high-bandwidth data stream into multiple parallel, lower-bandwidth sub-streams. Each sub-stream is independently modulated onto a closely spaced orthogonal subcarrier [20], forming the basis of our proposed solution.

In the field of radar and communication signal processing, suppressing inherent system interference and enhancing key performance metrics through optimization algorithms remains a core research objective. However, traditional channel estimation methods often exhibit limited performance in complex near-field channel environments, struggling to meet modern communication systems’ demands for high accuracy and robustness. To address this, Reference [21] proposes a low-complexity sparse adaptive channel estimation scheme driven by dynamic thresholds based on MIMO-FBMC technology, significantly reducing computational complexity and making it suitable for scenarios such as industrial big data communications. Reference [22] proposed the MBPD algorithm for near-field broadband XL-MIMO systems. By employing adaptive weighting and a multi-candidate evaluation mechanism, it effectively enhances the accuracy and robustness of channel estimation. In recent years, with the rapid development of industrial big data communications and massive antenna systems, multi-carrier phase coding (MCPC) signals have garnered widespread attention due to their immense potential in the field of radar-communication fusion.

Optimizing the side lobe level in pulse-compressed MCPC waveforms remains a formidable challenge. MCPC signals exhibit high side lobe issues after pulse compression, limiting their application in weak target detection. To address this issue, Reference [23] employed a Logistic chaotic sequence as the phase encoding for MCPC signals, proposing an MCPC-CS signal that combines chaotic coding with single-tone encoding. The MCPC-CS signal demonstrates excellent autocorrelation sidelobe suppression performance, successfully reducing sidelobe levels by over 3 dB compared to traditional MCPC signals, thereby providing an effective solution approach. Building upon existing research, this paper aims to further explore the sidelobe suppression potential of MCPC waveforms. First, we propose an AC-MCPC signal design method that combines time-domain chaotic coding with frequency-domain windowing. This method utilizes the randomness of chaotic sequences to optimize phase coding in the time domain while applying a hamming window in the frequency domain. It aims to significantly reduce autocorrelation sidelobe levels and enhance the system’s anti-interference capability in complex electromagnetic environments. To overcome the performance limitations of fixed-parameter window functions, we introduce a genetic algorithm [24] for intelligent optimization of the hamming window parameters embedded in the AC-MCPC waveform. Leveraging the global search capability of the genetic algorithm, we identified an optimal parameter combination surpassing standard configurations. Experimental results demonstrate that the AC-MCPC-g signal, refined through genetic algorithm optimization, achieves a 0.25 dB improvement in peak sidelobe ratio over the already low sidelobe performance of AC-MCPC.

The remainder of this paper is organized as follows. Section 2 details the AC-MCPC waveform design model. Section 3 focuses on the optimal waveform design for time sidelobe minimization. Section 4 presents simulation and experimental results on temporal sidelobe performance and provides a comprehensive analysis. Section 5 analyzes the performance of signals against intermittent sampling interference. Finally, Section 6 concludes the paper and summarizes the main findings.

## 2. AC-MCPC Waveform Design Models

**Figure 1:**
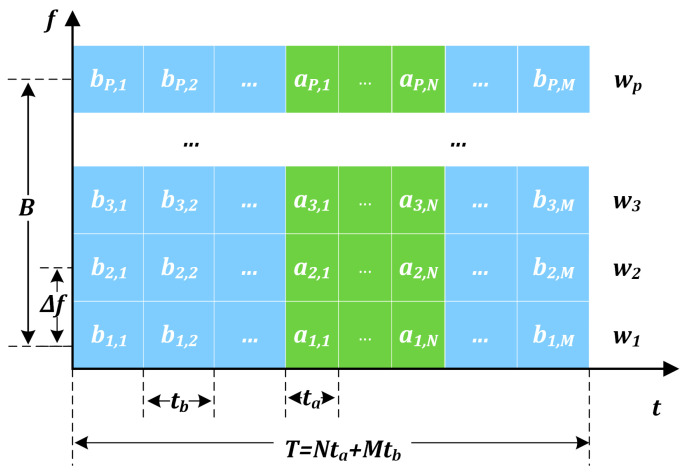
Time–frequency structure diagram of the AC-MCPC signal. Note: The ellipsis (⋯) indicates that certain subcarriers and symbols have been omitted for clarity.

The time–frequency representation of the AC-MCPC signal is shown in Figure 1, illustrating its composite structure. The signal consists of two main components: the MCPC chaotic coding segment and a single chaotic coding segment. These components are combined synergistically within the overall AC-MCPC chaotic coding framework.

The signal of AC-MCPC consists of *P* subcarriers, each containing *M + N* bits of phase coding. The variable $\omega_{p}$ in the time–frequency structure diagram of the AC-MCPC signal represents the weighted amplitude, $a_{p,n}$ is the phase encoding of the n th code slice on the p th subcarrier, a signifies a random single chaotic code, $t_{a}$ is the duration of a single code slice, $b_{p,m}$ stands for MCPC chaotic coding at time $t_{b}$, and B refer to the bandwidths. Phase coding with a single chaotic hybrid code is employed in the time domain, while a genetic algorithm is utilized in the frequency domain to optimize the parameters of the carrier weighting window function.

According to [25], the mathematical expression for the MCPC signal re-enveloping is given as follows:

(1) $$s(t)=\sum_{p=1}^{P}\sum_{m=1}^{M}\omega_{p}b_{p,m}rect[t-(m-1)t_{b}]\exp[j2\pi (p-1)\Delta f_{b}t].$$

The expression for the AC-MCPC signal is:

(2) $$\begin{array}{l} y(t)=\sum_{p=1}^{P}\sum_{m=1}^{X}\omega_{p}b_{p,m}\exp(j2\pi f_{p}t)rect[t-(x-1)t_{b}]\\ +\sum_{p=1}^{P}\sum_{n=x+1}^{N+X}\omega_{p}a_{p}\exp(j2\pi f_{p}t)rect[t-(n-1)t_{a}]\\ +\sum_{p=1}^{P}\sum_{m=n+x+1}^{M+N}\omega_{p}b_{p,m}\exp(j2\pi f_{p}t)rect[t-(m-1)t_{b}], \end{array}\;\mathrm{included}\mathrm{among}\mathrm{these},rect(t)=\left\{\begin{array}{c} 1,0\leq t\leq t_{b}\\ 0,other \end{array}\right..$$

In multicarrier phase coding, chaotic sequences are extensively used to generate chaotic two-phase codes. Logistic mapping produces sequences that exhibit optimal performance in signal identification and interference suppression. Therefore, this paper employs logistic mapping for the phase coding of signals.

The iterative expression for the logistic mapping [26] is as follows:

(3) $$x_{n+1}=1-2x_{n}^{2},x_{n}\in [-1,1]$$

$x_{n}$: Current state value, representing the value of the chaotic sequence at iteration n;
$x_{n+1}$: Next state value, representing the value of the chaotic sequence at iteration n + 1.
*n*: Number of iterations.

The function of Doppler shift, as derived from reference [27], is given by

(4) $$\chi (\tau ,\xi )=\int_{-\infty }^{+\infty }s(t)s^{*}(t+\tau )\exp(j2\pi \xi t)dt,$$

The ambiguity function for the MCPC signal is derived by combining the definition in (4) with the expression for the MCPC signal in (1), as follows:

(5) $$\begin{array}{l} \chi \left(\tau ,f_{d}\right)=\int_{-\infty }^{\infty }s(t)s^{*}(t+\tau )\exp(j2\pi f_{d}t)dt\\ =\int_{-\infty }^{+\infty }\sum_{p=1}^{P}\sum_{m=1}^{P}\omega_{p}u(t)\exp(j2\pi f_{p}t)\omega_{m}^{*}u{\left(t+\tau \right)}^{*}\exp[-j2\pi f_{m}(t+\tau )]\exp(j2\pi f_{d}t)dt\\ =\sum_{p=1}^{P}\sum_{m=1}^{P}\omega_{p}\omega_{m}^{*}\exp(-j2\pi f_{m}\tau )\int_{-\infty }^{+\infty }u(t)u{\left(t+\tau \right)}^{*}\exp[j2\pi (f_{p}-f_{m})t]\exp(j2\pi f_{d}t)dt. \end{array}$$

The ambiguity function of the AC-MCPC signal:

(6) $$\begin{array}{l} \chi \left(\tau ,f_{d}\right)=\int_{-\infty }^{\infty }y(t)y*(t+\tau )\exp(j2\pi f_{d}t)dt\\ =\int_{-\infty }^{+\infty }\sum_{p=1}^{P}\sum_{m=1}^{P}\omega_{p}u(t)\exp(j2\pi f_{p}t)\omega_{m}^{*}u{\left(t+\tau \right)}^{*}\exp[-j2\pi f_{m}(t+\tau )]\exp(j2\pi f_{d}t)dt\\ =\sum_{p=1}^{P}\omega_{p}^{2}b^{2}\exp(-j2\pi f_{m}\tau )\sum_{q=-(M-1)}^{M-1}\chi_{u}(\tau -qt_{b},f_{d})\sum_{M-1}^{M-\left|q\right|}b_{p,m}b_{p,m+\left|q\right|}^{*}\exp(j2\pi f_{d}mt_{b})\\ +\sum_{p=1}^{P}\omega_{p}^{2}a^{2}\exp(-j2\pi f_{n}\tau )\sum_{l=-(N-1)}^{N-1}\chi_{u}(\tau -lt_{a},f_{d})\sum_{N-1}^{N-\left|l\right|}a_{p,n}a_{p,n+\left|l\right|}^{*}\exp(j2\pi f_{d}nt_{a})\\ +\sum_{p=1}^{P}\sum_{m=1,p\neq m}^{M}\omega_{p}\omega_{m}^{*}b_{p}b_{m}^{*}\exp(-j2\pi f_{m}\tau )\sum_{q=-(M-1)}^{M-1}\chi_{u}(\tau -qt_{b},f_{p}-f_{m}+f_{d})\sum_{M-1}^{M-\left|q\right|}b_{p,m}b_{p,m+q}^{*}\exp(j2\pi f_{d}mt_{b})\\ +\sum_{p=1}^{P}\sum_{n=1,p\neq n}^{N}\omega_{p}\omega_{n}^{*}a_{p}a_{n}^{*}\exp(-j2\pi f_{n}\tau )\sum_{l=-(N-1)}^{N-1}\chi_{u}(\tau -lt_{a},f_{p}-f_{n}+f_{d})\sum_{N-1}^{N-l}a_{p,n}a_{p,n+l}^{*}\exp(j2\pi f_{d}nt_{a}). \end{array}$$

$\chi_{u}(\tau ,f_{d})$ is denoted as:

(7) $$\chi_{u}(\tau ,f_{d})=\left\{\begin{array}{l} \frac{\sin(\pi f_{d}(t_{b}-\left|\tau \right|))}{\pi f_{d}(t_{b}-\left|\tau \right|)}(1-\frac{\left|\tau \right|}{t_{b}})\exp[j\pi f_{d}(t_{b}-\tau )],\;\left|\tau \right|\leq t_{b}\\ 0,\;\left|\tau \right|>t_{b} \end{array}\right.$$

where $y^{*}(t+\tau )$ represents the impulse response coefficient of the matched filter, while $\chi_{u}(\tau ,f_{d})$ denotes the fuzzy function of y(t).

**Figure 2:**
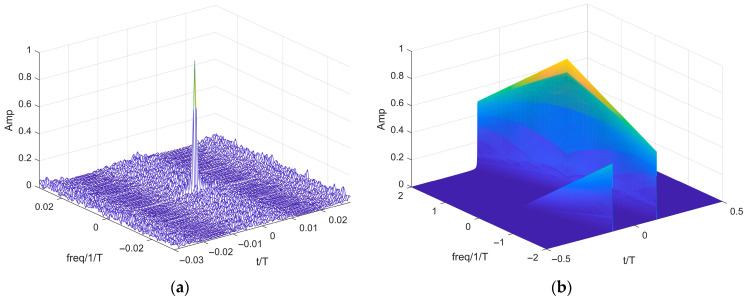
Three-dimensional blur function diagram of the signal. (**a**) Three-dimensional fuzzy function diagram of AC-MCPC signals; (**b**) three-dimensional fuzzy function diagram of the LFM signal.

To comprehensively evaluate the performance of the proposed AC-MCPC waveform, we conducted a core comparative analysis against traditional linear frequency-modulated signals, focusing on the characteristic differences in their fuzzy functions, as shown in Figure 2a,b.

As shown in Figure 2b, the main peak of the LFM signal’s ambiguity function exhibits a sloped profile, indicating the presence of a range-Doppler coupling effect. In contrast, the AC-MCPC signal depicted in Figure 2a displays an ideal “pin-shaped” ambiguity function contour. This structure is characterized by its energy being highly concentrated near the origin, while responses in other regions are rapidly suppressed to extremely low levels. This characteristic is crucial for modern radar systems operating in complex electromagnetic environments. In such scenarios, this waveform mitigates interference from dense targets while minimizing false alarms caused by high sidelobes, thereby enhancing target detection reliability. Consequently, the AC-MCPC waveform offers significant performance advantages over traditional LFM signals.

## 3. Optimal Waveform Design for Time Sidelobe Minimization

A critical preliminary step in our methodology is the empirical validation of the optimization capabilities of the genetic algorithm (GA) and the selection of the most appropriate window function for MCPC waveform design. To achieve this, we systematically evaluated several classical window functions: rectangular, hamming, hanning, blackman, and kaiser windows. In each evaluation, the window was combined with a Schroeder phase code to form the weighting factor for an MCPC signal. The resulting autocorrelation functions were then simulated to assess their sidelobe performance. Among all evaluated combinations, the pairing of hamming windows with Schroeder phase codes demonstrated the most outstanding side lobe suppression performance. Based on this finding, we ultimately selected this combination as the weighting function for the genetic algorithm optimization process.

The expression for the generalized hamming window function is:

(8) $$w_{n}={\left(a_{0}-a_{1}\cos\frac{2\pi n}{N}\right)}^{\alpha }$$

The genetic algorithm optimizes the parameters of the hamming window. The key parameter in waveform design is the hamming window coefficient α, $a_{0}$ and $a_{1}$. These coefficients are encoded as chromosomes within the genetic algorithm, with α, $a_{0}$ and $a_{1}$ constrained to the range $[0.1,0.9]$ and subject to the constraint that $a_{0}+a_{1}=1$.

The expression for the generalized hamming window function is transformed as follows:

(9) $$w_{n}={\left[a_{0}-(1-a_{0})\cos\frac{2\pi n}{N}\right]}^{\alpha }$$

To further enhance the side lobe suppression performance of AC-MCPC signals, this study employs a genetic algorithm to precisely optimize the parameters of the standard hamming window. The primary objective of this optimization is to minimize the peak side lobe level of the waveform. Accordingly, the fitness function of the algorithm is directly defined as the PSLR value of the AC-MCPC waveform.

Within the framework of genetic algorithms, the key parameters of the hamming window to be optimized are $[\alpha ,a_{0},a_{1}]$. These three parameters collectively determine the precise shape of the window function. We impose the linear constraint $a_{0}+a_{1}=1.$

The algorithm’s optimization process is as follows: For each individual in the population each parameter combination $[\alpha ,a_{0},a_{1}]$, the algorithm first constructs a corresponding custom hamming window function based on these parameters. This window function is applied to generate an AC-MCPC signal, and the performance of the resulting AC-MCPC-g waveform is evaluated by calculating its PSLR value. The PSLR value serves as the fitness score for each individual, with a lower PSLR indicating higher fitness. Through genetic operations such as selection, crossover, and mutation, the algorithm terminates upon reaching a preset maximum number of iterations. Finally, the set of parameters $[\alpha ,a_{0},a_{1}]$ identified during the evolutionary process that achieves the global minimum PSLR value for the AC-MCPC waveform is selected as the optimal solution. This optimal parameter set defines the hamming window used in the AC-MCPC-g signal, which exhibits enhanced sidelobe suppression performance and is ultimately proposed.

**Figure 3:**
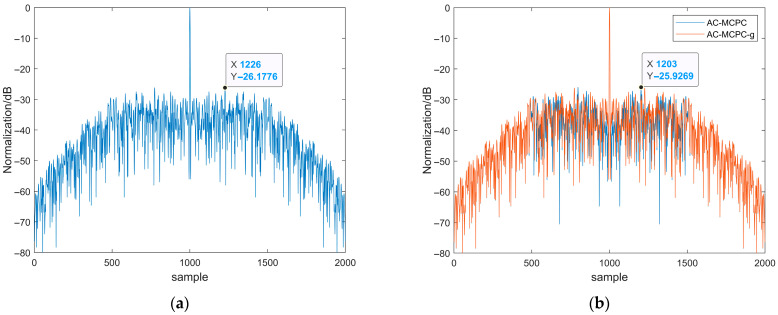
Autocorrelation function plot of the signal: (**a**) Autocorrelation comparison of AC-MCPC signals using genetic algorithms; (**b**) Comparison of AC-MCPC signal autocorrelation with or without genetic algorithm.

For clarity, we designate the carrier-weighted signal without genetic algorithm optimization as AC-MCPC, while the signal optimized using the genetic algorithm is referred to as AC-MCPC-g. Under the specified conditions of signal bandwidth B = 500 MHz and time width T = 1 μs, we conducted a comparative analysis of the performance of both signals. As shown in Figure 3, Figure 3a displays the autocorrelation function of the AC-MCPC-g signal. In contrast, Figure 3b visually compares the autocorrelation functions of both AC-MCPC and AC-MCPC-g. The figures clearly demonstrate that the genetically optimized AC-MCPC-g signal exhibits significantly lower temporal sidelobe levels than the unoptimized AC-MCPC signal. To validate the statistical reliability of this performance improvement, we repeated the experiment multiple times under identical conditions and recorded the sidelobe suppression values.

In this optimization process, the weight coefficients $[\alpha ,a_{0},a_{1}]$ of the genetic algorithm serve as core regulatory parameters, whose values directly determine the final side lobe suppression effect. Through systematic experiments with the objective of minimal side lobe suppression, we ultimately identified the optimal weight combination as α = 0.6, $a_{0}$ = 0.55, $a_{1}$ = 0.45.

Under conditions of bandwidth B = 500 MHz and pulse duration T = 1 μs, we evaluated the performance of the genetically optimized AC-MCPC waveform. As shown in Figure 3, the genetically optimized AC-MCPC-g waveform demonstrates superior autocorrelation sidelobe suppression compared to the waveform employing a traditional hamming window. Specifically, the autocorrelation sidelobe level of the hamming-weighted waveform is −25.93 dB, while the optimized AC-MCPC-g waveform further reduces it to −26.18 dB, achieving a 0.25 dB improvement. This result strongly demonstrates that the genetic algorithm can effectively reduce the autocorrelation time sidelobes of AC-MCPC signals, making it a superior optimization method compared to traditional window functions.

## 4. Time Sidelobe Simulation Experiments and Analysis

To ensure experimental accuracy and enable fair comparison of different waveform designs, we established a set of fixed simulation parameters: signal bandwidth B = 500 MHz, time width T = 1 µs. Under these unified conditions, we compared and analyzed the performance of seven signals: LFM signal, H-LFM signal (LFM processed with hamming window weighting), MCPC signal, H-MCPC signal (MCPC processed with combined hamming window and Schroeder initial phase weighting), the MCPC-CS signal proposed in [23], AC-MCPC signal, and the AC-MCPC-g signal—a more refined optimization strategy proposed herein.

**Figure 4:**
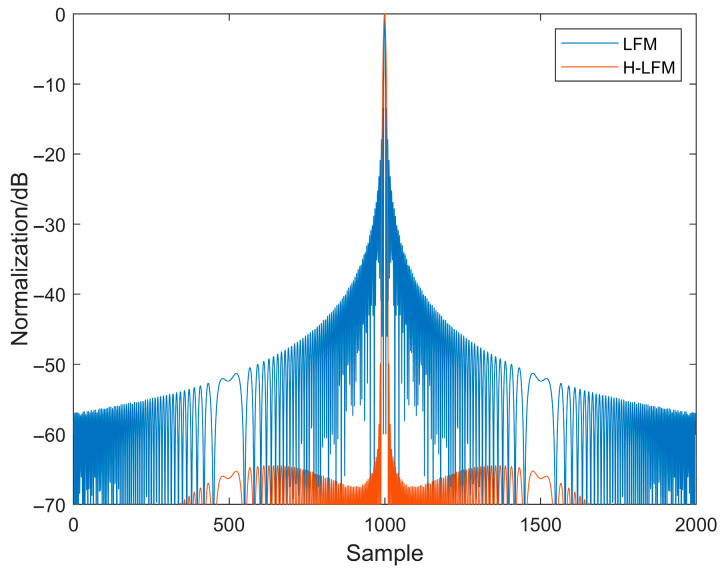
Autocorrelation of the two signals: H-LFM versus LFM.

**Figure 5:**
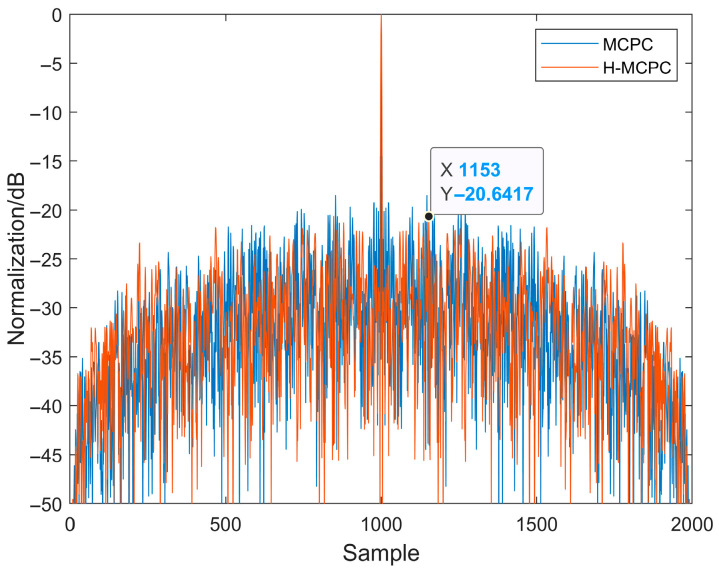
Autocorrelation of the two signals: H-MCPC versus MCPC.

**Figure 6:**
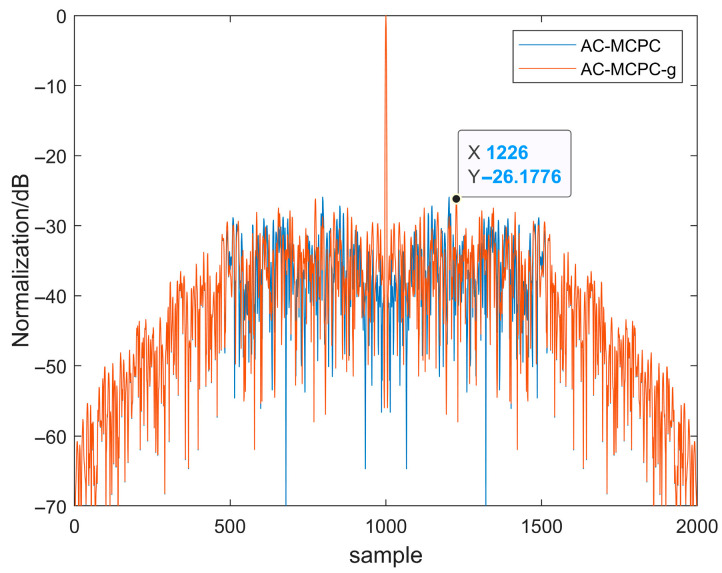
Autocorrelation of the two signals: AC-MCPC-g versus AC-MCPC.

**Figure 7:**
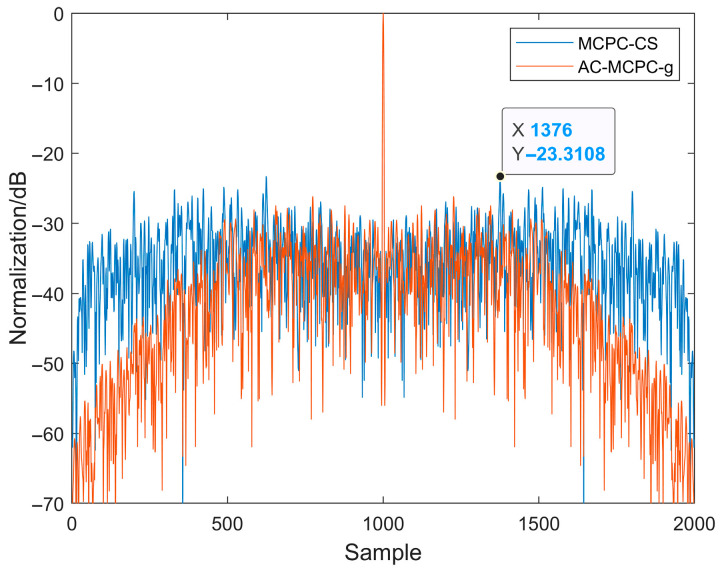
Autocorrelation of the two signals: AC-MCPC-g versus MCPC-CS.

Figure 4 compares the autocorrelation functions of linear frequency modulation (LFM) signals with and without windowing. As shown in Figure 4, applying a hamming window significantly reduces side lobe energy. Although side lobe energy is markedly reduced after windowing, the LFM signal exhibits poor interference resistance. Figure 5 compares the autocorrelation functions of MCPC and H-MCPC signals. A window application reduces the sidelobes of the MCPC signal. Figure 6 compares the autocorrelation functions of AC-MCPC and AC-MCPC-g signals. It is evident that the autocorrelation function of the AC-MCPC-g signal is slightly superior to that of the AC-MCPC signal. Through window function optimization, the AC-MCPC-g signal further reduces sidelobes by 0.25 dB. Figure 7 shows the autocorrelation comparison between the MCPC-CS signal and the AC-MCPC-g signal, where the AC-MCPC-g signal exhibits lower sidelobes than the MCPC-CS signal. Simulation results obtained on the MATLAB R2024a platform demonstrate that the proposed AC-MCPC-g signal exhibits outstanding performance in autocorrelation side lobe suppression, with side lobe levels lower than those of several other signals.

To evaluate the autocorrelation performance of a signal, we use the peak-to-side lobe ratio (PSLR) metric. A lower PSLR value indicates better signal autocorrelation characteristics and enhanced overall system performance.

As indicated in Reference [23], the PSLR expression is:

(10) $$\mathrm{PSLR}=\frac{\underset{k\in \text{side-lobe}}{\max}\left|A(k)\right|}{\underset{k\in main\text{-lobe}}{\max}\left|A(k)\right|}$$

where A(k) is the discrete expression of the autocorrelation function.

Table 1 presents a comparison of the autocorrelation performance between AC-MCPC-g signals and AC-MCPC signals, as well as other signals, under three sets of different parameter configurations. The first signal set features a bandwidth B = 500 MHz and a time width T = 1 µs. The second signal set has a bandwidth B = 300 MHz and a time width T = 10 µs. The third group has a bandwidth B = 64 MHz and a time width T = 128 µs.

To effectively suppress the autocorrelation time-side lobe level of multi-carrier phase-coded (MCPC) radar waveforms, this section conducts a comparative analysis of autocorrelation side lobe suppression performance across seven radar signals under three distinct bandwidth and time-width parameters. This evaluation validates the effectiveness of the proposed two-stage progressive signal design strategy for system assessment, with results presented in Table 1.

Simulation data strongly support the proposed signal design approach. In the first stage, the AC-MCPC signal—generated by combining chaotic coding with hamming window weighting—achieved a significant peak side lobe level reduction exceeding 10 dB compared to the baseline MCPC signal. Furthermore, the AC-MCPC signal demonstrated a peak side lobe level reduction of over 2 dB relative to the MCPC-CS signal. In the second stage, building upon the AC-MCPC signal, the window function parameters were intelligently optimized using a genetic algorithm. This fine-tuning successfully extracted an additional 0.25 dB PSLR gain on top of the already low sidelobes of the AC-MCPC signal. This result confirms the effectiveness of the second-stage fine-tuning strategy. The efficacy of the two-stage progressive strategy is thus comprehensively validated.

## 5. Performance Analysis of Intermittent Sampling Interference Resistance

To systematically evaluate the effectiveness of the proposed AC-MCPC signal against intermittent sampling forwarding interference, this section designs a series of comparative simulation experiments. Experiments are conducted under strictly controlled parameters: carrier frequency of 35 GHz, signal bandwidth of 300 MHz, sampling rate of 600 MHz, pulse width of 6 μs, and the pulse repetition period is 60 μs. For fairness, all AC-MCPC, MCPC, and MCPC-CS signals in the comparison were uniformly subjected to a hamming window for side lobe suppression. The experiments employed a signal-to-noise ratio (SNR) of −3 dB and a signal-jamming ratio (SJR) of −6 dB. Pulse compression peak height served as the core metric. All data were statistically averaged over 500 independent Monte Carlo simulations to ensure reliability.

**Figure 8:**
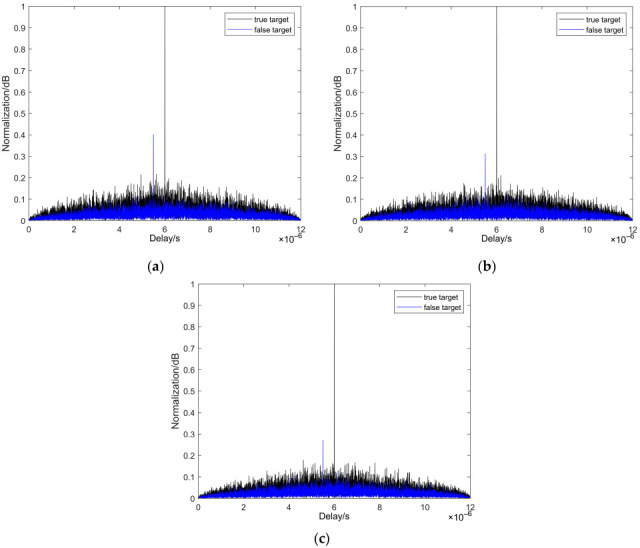
Performance of different signal countermeasures against intermittent direct forwarding interference: (**a**) MCPC intermittent sampling direct forwarding interference suppression effect; (**b**) MCPC-CS intermittent sampling direct forwarding interference suppression effect; (**c**) AC-MCPC intermittent sampling direct forwarding interference suppression effect.

**Figure 9:**
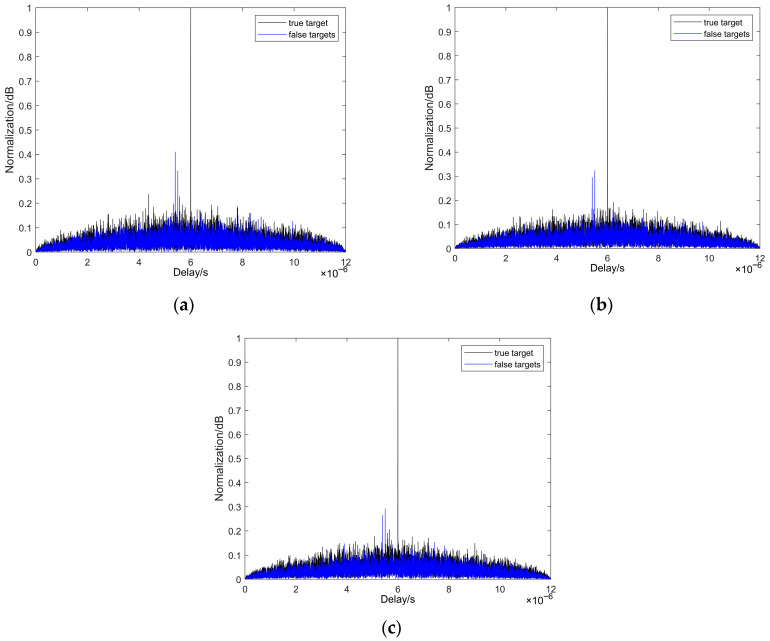
Performance of different signal countermeasures against intermittent sampling replication forwarding interference: (**a**) MCPC interference suppression effect against intermittent sampling replication forwarding; (**b**) MCPC-CS interference suppression effect against intermittent sampling replication forwarding; (**c**) AC-MCPC interference suppression effect against intermittent sampling replication forwarding.

To evaluate performance against two typical ISRJ patterns, we conducted comparative simulations. Figure 8 presents results for the anti-intermittent sampling direct forwarding interference scenario, while Figure 9 corresponds to the anti-intermittent sampling repeated forwarding interference scenario. Both figures compare the performance of AC-MCPC, MCPC-CS, and MCPC signals. The results clearly demonstrate that in both environments, the AC-MCPC signal exhibits superior false target suppression capability, with slightly enhanced anti-jamming robustness compared to MCPC and MCPC-CS signals.

To evaluate the performance of three signals anti-interrupted sampling direct jamming, comparative experiments were conducted in this paper. As shown in Figure 8, the results clearly demonstrate the performance advantage of the AC-MCPC signal against ISDJ. Specifically, under the condition of main lobe (true target) amplitude normalization, Figure 8a indicates that the traditional MCPC signal generates strong false targets with peaks as high as 0.4 dB in the ISDJ environment, severely compromising the reliable detection of true targets. Figure 8b shows that the optimized MCPC-CS signal exhibits improved performance, suppressing false target peaks by 0.3 dB. Most critically, the results in Figure 8c prove that the proposed AC-MCPC signal exhibits outstanding anti-interference capability, successfully suppressing false target peaks below 0.3 dB, outperforming the previous two signals in interference resistance.

To further validate the robustness of AC-MCPC signals, this study evaluated the performance of three signal types under adversarial anti-interrupted sampling repeater jamming. As shown in Figure 9, the results clearly demonstrate the performance advantage of the AC-MCPC signal against intermittent sampling repeat forwarding interference. Under identical test conditions, Figure 9a indicates that the traditional MCPC signal exhibits significant false target peaks at approximately 0.33 dB and 0.4 dB. In contrast, Figure 9b shows that the optimized MCPC-CS signal achieves significant improvement, suppressing the peak to approximately 0.31 dB. Most notably, Figure 9c demonstrates that the proposed AC-MCPC signal exhibits the strongest suppression capability, successfully reducing all false target peaks below 0.3 dB. In summary, the AC-MCPC signal consistently demonstrates superior false target suppression capability in both anti-interrupted sampling direct jamming and anti-interrupted sampling repeater jamming scenarios, exhibiting significantly enhanced anti-interference robustness compared to both MCPC and MCPC-CS waveforms.

**Figure 10:**
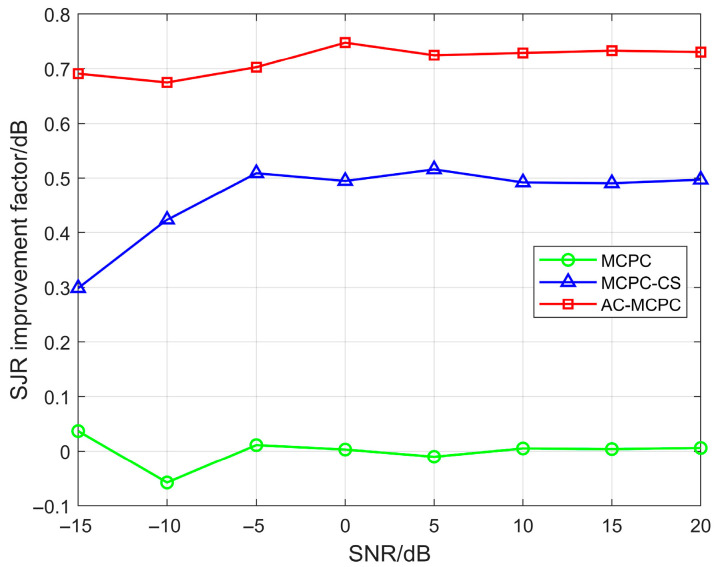
Simulation comparison of SJR = −6 dB improvement factor at different signal-to-noise ratios.

We conducted a comparative analysis of the SJR improvement factors for three signals under varying SNR conditions. As shown in Figure 10, under identical parameter settings, the AC-MCPC signal proposed in this paper consistently demonstrates superior interference resistance compared to both the MCPC and MCPC-CS signals. Specifically, the SJR improvement factor of the AC-MCPC signal increased by 0.25 dB compared to the MCPC-CS signal and by 0.74 dB compared to the traditional MCPC signal. This demonstrates that the AC-MCPC signal not only outperforms MCPC-CS and MCPC in sidelobe suppression capability but also exhibits slightly superior interference resistance relative to both MCPC-CS and MCPC signals.

## 6. Conclusions

This article presented an innovative AC-MCPC-g signal to address the sidelobe suppression challenge in radar waveform design. The design of this signal involved a two-step optimization process. First, we constructed the AC-MCPC signal as a foundation. By incorporating chaotic encoding in the time domain and a hamming window in the frequency domain, this foundational signal achieved a significant sidelobe suppression of over 10 dB compared to the basic MCPC signal, and an approximately 2.4 dB reduction compared to the MCPC-CS signal. On this basis, to further explore performance limits, we proposed the AC-MCPC-g signal. The core innovation of AC-MCPC-g lay in replacing the fixed hamming window in the AC-MCPC frequency domain with a weighted window function optimized via a genetic algorithm. By finely tuning the optimization parameters, AC-MCPC-g ultimately achieved an additional reduction of 0.25 dB beyond the already low sidelobes of AC-MCPC. Secondly, regarding anti-jamming performance, we tested the AC-MCPC signal against benchmark signals in an identical ISRJ environment. Simulation results showed that in suppressing false targets generated by ISRJ, the AC-MCPC signal exhibited superior performance compared to the MCPC and MCPC-CS signals.

However, this study still has certain limitations, pointing the way forward for future work. First, regarding parameter adaptability, the bandwidth and time width of existing AC-MCPC signals are both preset values, lacking mechanisms for dynamic perception and adaptive adjustment to real-time electromagnetic environments. This limits their application flexibility and robustness in complex, dynamic scenarios. Second, at the optimization algorithm level, this study primarily employs traditional weighting techniques such as the hamming window. While effective, these methods have relatively limited optimization capabilities and fail to fully integrate cutting-edge algorithms like artificial intelligence to achieve deep performance mining. Moreover, the current optimization framework suffers from a single-objective limitation, focusing primarily on the isolated metric of sidelobe suppression. Practical engineering systems often require balancing multiple objectives, such as anti-interference performance and target resolution, making single-objective optimization inadequate for comprehensive requirements. Finally, anti-interference performance, a critical indicator of system practicality, has not been explicitly incorporated into the optimization objectives, adversely affecting the system’s overall performance and robustness. Specifically, this research will investigate real-time dynamic parameter adjustment mechanisms, construct multi-objective optimization models incorporating metrics like interference resistance and resolution, and develop efficient intelligent solution algorithms. Through these efforts, the goal is to comprehensively enhance the overall performance and environmental adaptability of AC-MCPC signals, driving their widespread application and value realization in practical engineering systems.

## Figures and Tables

**Table 1:** Autocorrelation performance in dB.

Signal	Parameters		
	500 MHz/1 µs	300 MHz/10 µs	64 MHz/128 µs
LFM	−13.48	−13.47	−13.46
H-LFM	−49.77	−49.66	−49.64
MCPC	−15.51	−13.90	−13.60
H-MCPC	−20.64	−27.07	−30.28
MCPC-CS	−23.63	−29.69	−33.08
AC-MCPC	−25.93	−31.95	−35.22
AC-MCPC-g	−26.18	−32.25	−35.48

## Data Availability

The original contributions presented in this study are included in the article. Further inquiries can be directed to the corresponding author.
